# Inverse Poly-High Internal Phase Emulsions Poly(HIPEs) Materials from Natural and Biocompatible Polysaccharides

**DOI:** 10.3390/ma13235499

**Published:** 2020-12-02

**Authors:** Giuseppe Tripodo, Enrica Calleri, Cinzia di Franco, Maria Luisa Torre, Maurizio Memo, Delia Mandracchia

**Affiliations:** 1 Department of Drug Sciences, University of Pavia, Viale Taramelli 12, 27100 Pavia, Italy; giuseppe.tripodo@unipv.it (G.T.); enrica.calleri@unipv.it (E.C.); marina.torre@unipv.it (M.L.T.); 2 CNR-IFN Bari, Via Amendola 173, 70126 Bari, Italy; cinzia.difranco@uniba.it; 3 Department of Molecular and Translational Medicine, University of Brescia, Viale Europa 11, 25123 Brescia, Italy; maurizio.memo@unibs.it

**Keywords:** inulin, chitosan, glutaraldehyde, UV, crosslinking, HIPE, wound dressing, hydrogel, high internal phase emulsion, polysaccharide

## Abstract

This paper shows one of the few examples in the literature on the feasibility of novel materials from natural and biocompatible polymers like inulin (INU) or glycol chitosan (GCS) templated by the formation of *o*/*w* (inverse) high internal phase emulsion (HIPE). To the best of our knowledge, this is the first example of inverse polyHIPEs obtained from glycol chitosan or inulin. The obtained polyHIPEs were specifically designed for possible wound dressing applications. The HIPE (pre-crosslinking emulsion) was obtained as inverse HIPE, i.e., by forming a cream-like 80:20 *v/v o*/*w* emulsion by using the isopropyl myristate in its oil phase, which is obtained from natural sources like palm oil or coconut oil. The surfactant amount was critical in obtaining the inverse HIPE and the pluronic F127 was effective in stabilizing the emulsion comprising up to 80% *v*/*v* as internal phase. The obtained inverse HIPEs were crosslinked by UV irradiation for methacrylated INU or by glutaraldehyde-crosslinking for GCS. In both cases, inverse poly-HIPEs were obtained, which were physicochemically characterized. This paper introduces a new concept in using hydrophilic, natural polymers for the formation of inverse poly-HIPEs.

## 1. Introduction

To treat skin wounds due to acute (burns, accidents) or chronic (sores) events we are still looking for effective biomaterials that can act, after application on the injured tissue, in different ways. These may include, e.g., antibacterial activity, exudate absorption, tissue regeneration, and drug release. An optimal biomaterial for wound dressing could also be “inert” and allowed to exert its function by introducing specific molecules or functionalities. Another important aspect is the control of the final shape and a tailorable internal structure with predictable internal interconnections and porosity. The former behaviors are mostly dependent on the material(s) used and the latter on the production technique applied [1,2].

In the last few years, there has been growing interest in templating the internal structure of biomaterials by emulsion techniques such as the formation of high internal phase emulsions (HIPEs). This technique is one of the best ways to template the internal structure in terms of porosity and interconnections, also allowing control over the final shape [3,4]. Since HIPEs are composed (e.g., *v*/*v*) of internal phase by up to 90%, the external phase will be highly stretched in surrounding the internal phase droplets [5]. This brings serious problems for the stabilization of the system and even small variation in the composition of the HIPEs could lead to breaking of the emulsion (most commonly, phase inversion or separation). In literature, often HIPEs are formulated as *w*/*o* emulsion also due to the high amount of available hydrophobic liquid-monomers (at r.t.) that can be used as oil phase to be used as external phase to be crosslinked/polymerized after the HIPE formation. Examples of commonly used monomers are different acrylates, divinyl benzene, and styrene [6,7]. These monomers, upon polymerization, lead to polymerized and even crosslinked materials (polyHIPEs) showing remarkable hydrophobic behaviors on both the surface and internal structure.

The stabilization of these emulsions (*w*/*o*) is often performed by using low HLB (Hydrophilic Lipophilic Balance) value surfactants such as sorbitan monooleate or PEG-PPG-PEG co-polymers, however in the last few years, the so-called Pickering emulsions were also proposed. In these systems, the use of the surfactant is substituted with solid micro/nanoparticles that stabilize the *o*/*w* interphase [8,9]. Examples of these micro/nanostructures are denatured proteins, silica, or carbon nanotubes [10,11]. O/W HIPEs are less explored, probably due to the fact that stabilizing such emulsions is still more difficult. Due to the high number of developed *w*/*o* HIPEs, the *o*/*w* HIPEs are called inverse HIPEs. Lately, some example of inverse HIPEs have been shown in the literature [12], but still few with respect to *w*/*o* HIPEs. From a pharmaceutical/biomedical point of view, inverse polyHIPEs have the advantage of exposing a hydrophilic material that often shows more affinity to living tissue to the physiological environment. These premises and our experience in formulating *w*/*o* HIPEs brought our research to focus on using the characteristic HIPE templating in producing biomaterials for wound dressing. In particular, it has been thought that dispersing hydrophilic polymer(s) in water would produce an external phase that upon crosslinking, would result in a biomaterial that on one side, has the chemical, physical, and biologic properties of the used polymer and on the other, would be templated by the HIPE formation. Moreover, depending on the polymer water-dispersion concentration (as it happens with many hydrophilic polymers), the viscosity of the system could be controlled. In this way, according to the Stokes-Einstein equation, the viscosity of the continuous phase can be exploited in stabilizing the emulsion. Here, the glycol chitosan was used to prepare novel inverse HIPEs crosslinked by glutaraldehyde to form new polyHIPEs. Glycol chitosan was selected because of its intrinsic antimicrobial activity, which would prevent wound infections, as well as for its high hydrophilicity, which can promote exudate absorption. Moreover, considering the presence of primary amine groups, it could be exploited for post-production functionalization of the material, also predicting the possibility of loading hydrophilic proteic and peptidic drugs [13]. In 2018, our research group developed a new hydrogel composed of glycol chitosan crosslinked by PEG-diglycidyl ether in physiological conditions. This material exhibited pronounced antibacterial properties and a high affinity with water (exudate absorption). Moreover, it exhibited a remarkable pro-angiogenic activity, which is an important feature in the wound healing process [14]. In this way, angiogenesis promotion is a fundamental aspect in wound healing and its role is of growing interest in the scientific community. Mathematical models were also developed to explain the process [15,16,17].

At the same time, methacrylated Inulin [18,19] was also used to form an external phase to produce an inverse HIPE and the consequent polyHIPE, by UV irradiation. Inulin was selected as an example of biocompatible and hydrophilic polymer with versatile behaviors, such as a simple chemical functionalization, which can be exploited either pre- or post-production. Furthermore, vitamin E functionalized inulin as well as native inulin, showing pronounced pro-angiogenic activity [20,21]. Moreover, due to the reduced molecular weight (Mw) of inulin, usually around 5000 Da, its contribution to the viscosity of the external phase is reduced and also, it produces transparent dispersions, which make inulin an optimal polymer for obtaining hydrogels upon UV irradiation. Moreover, by considering the UV-induced radical mechanism for methacrylated Inulin crosslinking, a relatively low viscosity of the dispersion would favor the spreading of the radicals within the forming material. Based on these premises, this work shows the feasibility of two different inverse polyHIPEs from two different polymers by applying two different crosslinking approaches.

Furthermore, when using these systems for biomedical applications, it is fundamental to employ chemicals that should be as much as possible biocompatible and, eventually, easy to remove (if needed) from the final material. For this reason, in this work, isopropyl myristate was selected as an oil phase partially derived from natural sources (myristic acid), which has different pharmaceutical and cosmetic applications since it is used for topical applications and has, for example, moisturizing and penetration enhancing properties [22]. At high doses, comedogenic activity has been reported in some subjects [23]. Isopropyl myristate is immiscible with water but miscible with ethanol, which is an important feature to allow its easy removal from the material (if needed) and also strongly reduces eventual microbiological contamination of the final material. The so-conceived inverse HIPEs, comprising a water dispersion of the two proposed polymers and the isopropyl myristate as oil phase, were stabilized by a PEG-PPG-PEG surfactant at a high HLB value (hydrophilic surfactant). Based on the above premises, in this work, two new materials with an internal structure templated by the HIPE technique were prepared as specifically designed for wound dressing applications. To the best of our knowledge, this is the first example of polyHIPEs obtained from glycol chitosan or inulin.

## 2. Materials and Methods

### 2.1. Materials

Glycol chitosan (GCS), Inulin from Dahlia tuber (INU, Mw ~5000 Da), methacrylic anhydride (MA), triethylamine (TEA), glutaraldehyde (GLU), Anhydrous N,N-dimethylformamide (DMF) 99.9%, isopropyl myristate, and Tween 20 were purchased from Sigma-Aldrich (Milan, Italy) and used as received. Diethyl ether, acetone, and ethanol were purchased from Merck (Darmstadt, Germany). Further, 30 mL Syringe PP/PE without a needle luer lock tip and female luer coupler in polypropylene were purchased from Sigma-Aldrich (Milan, Italy). The water used in this study was double deionized water (DDW), obtained by a Milli-q system from Millipore.

### 2.2. Apparatus

An oven TCN30 (ArgoLab, Carpi, Modena, Italy) with forced ventilation was used for polyHIPEs polymerization.

UV irradiation of INU-MA HIPE was accomplished with a “Polymer” reactor (HeliosItalquartz, Milan, Italy) provided with a UV lamp of 125 W, with an emission range 250–364 nm. Its emission peak was 310 nm.

Centrifugations were performed with a Beckman Avanti 30 (Beckman, Milano, Italy) equipped with a temperature control.

Lyophilizations were performed by a Christ Alpha 1–4 LSC instrument at −59 °C and 0.016 mbar. ^1^H-NMR were acquired with a Varian Mercury 300 MHz instrument (Varian, Segrate, Italy).

SEM images of the surface and the cross-section of the lyophilized polyHIPEs were acquired by a ∑igma Zeissfield emission scanning electron microscope (FE-SEM) (Zeiss, Milan, Italy). Prior to the analysis, the samples were stuck on stubs with a carbon adhesive disc and subsequently subjected to coating by a 2 nm layer of palladium by using an electron beam evaporator. The SEM probing e-beam was set at an acceleration voltage of 3 kV; 30μm slit aperture and the in-lens detector were used.

### 2.3. Methods

#### 2.3.1. HIPE Preparation from GCS and INU-MA Polymers

HIPEs were prepared from two different biodegradable polymers, glycol chitosan (GCS) and methacrylated Inulin (INU-MA); the latter was synthesized using a previously reported procedure [1]. Briefly, 1 g of INU was dissolved in 14 mL of anhydrous DMF under N_2_. After complete solubilization, suitable amounts of TEA, as a catalyst, and MA were added according to the following ratio, with “RU” representing Repeating Unit:

R_1_ = Moles of MA/Moles of RU INU = 0.5

R_2_ = Moles of TEA/Moles of MA = 0.5.

The reaction mixture was maintained at 25 °C under stirring and under N_2_ for 20 h. After this time, the product was recovered by precipitation in a mixture of Et_2_O/acetone (2:1) and centrifuged for 5 min at 4.000 rpm and 4 °C. The product was then washed several times with the same solvents and then freeze-dried. The derivatization degree (DD mol/mol%) was 45% mol, which is in agreement with theorical data and was calculated from ^1^H-NMR as the ratio between the integral of the peaks at 5.70 and 6.08 ppm (MA, s, 2H) and the integral of the peaks at 3.5–4.0 ppm (fructose ring, m, 7H).

HIPEs were prepared following previously reported methods with modifications [2,3]. Here, we used different polymer concentrations in order to evaluate their influence on the HIPE formation process.

First, studies were carried out to select the surfactant leading a stable emulsion, which was chosen between Tween 20 and Lutrol F127, to evaluate the effect of their different HLB and molecular weights. Based on the obtained results, which are summarized in Table 1, we selected Lutrol F 127 for further studies.

Isopropyl myristate was selected as the oil phase and the oil/water ratio of 4/1 was employed for all HIPE preparations (also, propylene carbonate was employed without success).

**Figure 1:**
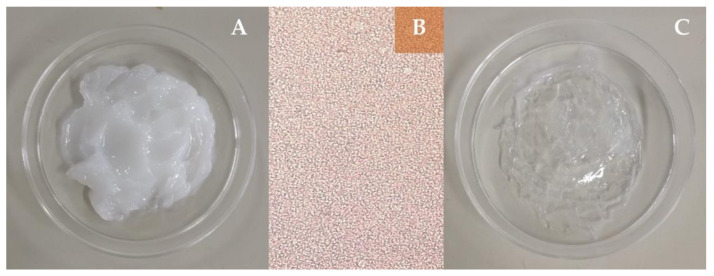
(**A**) Optical image of a typical G-series HIPE; (**B**) optical micrographs (40×) of HIPE in the presence of the Sudan Red G; and (**C**) optical image of G-series polyHIPE chemically crosslinked by glutaraldehyde.

In particular, for G-series HIPEs, GCS was solubilized in double distilled water at different concentrations of 1% (*w*/*v*) or 2% (*w*/*v*), called G1 and G2, respectively, and in two separate 2-neck round bottom glass flasks, each stirred at room temperature until dissolved. An appropriate amount of Lutrol F127, corresponding to 15% (*w*/*v*), was added to each flask and stirred at room temperature until completely dissolved. Then, each flask was placed on a stirrer equipped with a PTFE paddle and stirred at 300 rpm. Isopropyl myristate (16 mL) was added drop by drop over 5 min to each aqueous polymer solutions to ensure the emulsion formation. After the adding of the oil phase was completed, the stirring speed was increased up to 400 rpm. The emulsion was stirred under N_2_ flow at room temperature for 1 h, which is the time required to obtain a cream-like HIPE, as shown in Figure 1.

**Figure 2:**
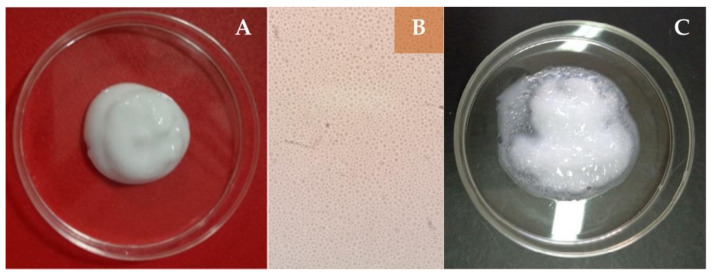
(**A**) Optical image of a typical I-series HIPE; (**B**) optical micrographs (40×) of HIPE in the presence of the Sudan Red G; and (**C**) optical image of I-series polyHIPE from UV crosslinking.

For I-Series, INU-MA conjugate was placed in 2-neck round bottom glass flasks and solubilized in double distilled water at different concentrations of 6% (*w/v*), 8% (*w*/*v*), or 10% (*w*/*v*) and the obtained HIPE were called I1, I2, and I3, respectively. Each solution was stirred at room temperature until dissolved. Then, Lutrol F127 at 15% (*w*/*v*) concentration was added to each flask and stirred at room temperature until completely dissolved. Each flask was placed on a stirrer equipped with a PTFE paddle and stirred at 300 rpm. Isopropyl myristate (16 mL) was added drop by drop over 5 min to each aqueous polymer solution to ensure emulsion formation. After the adding of the oil phase was completed, the stirring speed was increased to 400 rpm. The emulsion was stirred under N_2_ flow at room temperature for 1 h, which is the time required to obtain a viscous white HIPE, Figure 2.

Table 2 summarizes the general HIPE compositions for G-series (made from GCS) and I-series (made from INU).

#### 2.3.2. PolyHIPE Preparation from GCS Polymer

The polyHIPEs were prepared by the crosslinking reaction with glutaraldehyde (GLU), at different concentrations of the O/W emulsions characterized by a large volume of internal phase (G-series HIPEs). General compositions of HIPE are reported in Table 2. In particular, for each G samples, G1 and G2, three different GLU concentrations were used, 50, 100, or 200 microliters, in order to evaluate the effect of different crosslink degrees on the swelling behavior of polyHIPEs. The typical synthesis was as follows: in order to polymerize the external phase of the G-series HIPEs (to obtain the polyHIPE materials), the emulsions were transferred to a PE Syringe connected by a female luer coupler to a second syringe pre-filled with an exact amount of GLU. Using a “syringe-to-syringe” method, HIPEs and GLU were mixed by an alternate extrusion from one syringe to the other (10 extrusion processes). The optimal number of extrusions was visually established by using a oil-soluble dye until colour uniformity of the emulsion was reached. The mixed material was then rapidly transferred onto a petri plate and modelled as a uniform layer. All materials were placed in an oven at 40 °C for 24 h. Each sample was then washed in EtOH (3 times × 100 mL) in order to remove the unreacted GLU and the oil phase. After the washing step, the materials were freeze dried to remove residual water.

#### 2.3.3. PolyHIPE Preparation from INU-MA Polymer

The polyHIPE materials were prepared by the UV polymerization reaction of the o/w emulsions characterized by a large volume of internal phase (I-series HIPEs). The general compositions of HIPE are reported in Table 2. In particular, three different concentration of INU-MA were used, 6%, 8%, or 10% (*w*/*v*), to obtain I1, I2, and I3 polyHIPEs, respectively, in order to evaluate the effect of different polymer concentrations on the crosslink degree and consequently, on the morphology and swelling behaviour of polyHIPEs. The typical synthesis was as follows: in order to polymerize the external phase of the I-series HIPEs (to obtain the polyHIPE materials), the emulsions were transferred to petri plates, modelled as a uniform layer, and irradiated for 2 h without any photo-initiator. After the irradiation time, the obtained polyHIPEs were recovered and each sample was then washed in EtOH (3 times × 100 mL) in order to remove the not crosslinked materials and the oil phase. After the washing step, the materials were freeze dried to remove residual water and characterized.

#### 2.3.4. Water Uptake Studies in Water

The water uptake studies on PolyHIPEs were performed in double-distilled water at 37 °C for 24 h. In particular, the precisely weighed amounts of the dried polyHIPEs (≈30 mg) were put in a glass tube, which was open in the lower part and was provided with a support glass filter of 10 mm in diameter and a G2 porosity. The tubes were put in pre-warmed water and the samples were left to swell. After the established time, the tubes were allowed to percolate the excess water at atmospheric pressure; then, the tubes were placed inside a centrifuge tube and centrifuged for 5 min at 3000 rpm. Water uptake was calculated as q = Ws/Wd, where Ws is the weight of the swollen polyHIPE, while Wd is the weight of the dry sample. The reported values are the mean of three measurements (*n* = 3) for each sample and are in accordance with a standard error of 5%. The statistical significance of the differences between the counts was determined by Student’s t-test for unpaired data. Means ± Standard Deviation (SD) were evaluated for all the parameters.

## 3. Results and Discussion

### 3.1. HIPE Feasibility Studies

To produce the HIPEs (starting cream-like emulsions) suitable to be crosslinked and used as final materials for wound dressing, different oil phases, such as internal phases, were evaluated. Namely, isopropyl myristate and propylene carbonate were tested as oil-like water-immiscible phases. Only isopropyl myristate was suitable for the HIPE formation. The tested immiscible phases for the HIPE preparation were propylene carbonate and isopropyl myristate. Propylene carbonate is partially miscible with water and for this reason, it was first saturated with water. It was used since it is biocompatible and easy to eliminate from the final material because it can be removed by both polar and non-polar solvents [24]. Furthermore, its density is close to that of water, so emulsion stability was maximized. Although conceptually suitable for HIPE preparation, in this work, we were not able to form the emulsion, probably due to the partial miscibility with water, which strongly reduced the activity of the used surfactants.

In this work, we also evaluated the ability of two hydrophilic nonionic surfactants with different HLB and molecular weight values to form HIPE. Isopropyl myristate at 80% (*v*/*v*) was used as the oil (internal) phase, Table 1.

Tween 20 (HLB 16.7, Mw 1228 Da) was ineffective in forming/stabilizing the HIPE. Even if the cream-like emulsion was obtained for surfactant concentrations higher than 25% *w*/*v*, the HIPE emulsions broke (coalescence/phase separation) after a few minutes.

On the other hand, a PEG-PPG-PEG co-polymer surfactant, Lutrol F127^®®^ (HLB 23, Mw 12,600 Da), was more suitable for the preparation of HIPEs, probably due to its higher hydrophilicity and molecular weights, which can aid in enhancing emulsion stability. In fact, at a 15% (*w*/*v*) concentration, it gives a stable emulsion. Higher concentrations gave the same results in term of stability, but the viscosity of the dispersions increased by increasing the concentrations, leading to systems being difficult to handle. For these reasons, for the preparation of the HIPEs, Lutrol F127 at 15% (*w*/*v*) was selected as the most suitable surfactant since it was able to form and stabilize emulsions containing up to 80% *v/v* of internal phase. All prepared HIPEs were cream-like white emulsions that were stable for more than 24 h at r.t. (Figure 1 and Figure 2).

Also, the used surfactants had different chemical structures; one was a polysorbate (Tween 20) and the other one was a PEG-PPG-PEG co-polymer. In another study, in which a *w*/*o* HIPE was produced, two surfactants chemically related to those that were used in this paper were tested [25]. Also, in this case, the sorbitan derivative failed to stabilize the emulsion, while the PEG-PPG-PEG co-polymer was suitable for the purpose. In particular, in the referred paper, it was explained that the PEG-PPG-PEG co-polymer surfactant would be more effective in stabilizing the emulsion due to the PEG and PPG flexible polymeric chains interpenetrating the internal and external phases, respectively. In the case of the *o*/*w* HIPEs proposed here, this could be even more true due to the high hydrophilic behaviours of the two PEG chains (the two external segments of the tri-block copolymer), which are in strict contrast to the PPG central chain, showing a great affinity to the oil phase. In other papers, the Triton X-305 (HLB-17.3) was also successfully used for obtaining an inverse HIPE as well as Tween 60 [12,26].

### 3.2. PolyHIPEs Preparation and Characterization

**Scheme 1:**
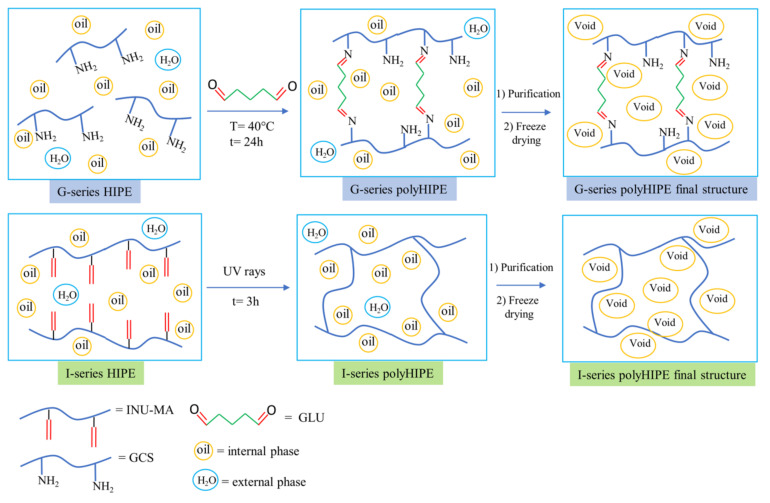
Schematic representation of G-series and I-series polyHIPE formation, describing from left to right: HIPE formation, polyHIPE formation (crosslinking), and polyHIPE purification and freeze drying.

Scheme 1 reports the procedure used to obtain the polyHIPE from GCS or INU-MA polymers. Table 2 summarizes the general compositions of I-series HIPEs and G-series HIPEs and the crosslinking agent used to obtain polyHIPEs material. See experimental Section 2.3.2 and Section 2.3.3 for details.

**Figure 3:**
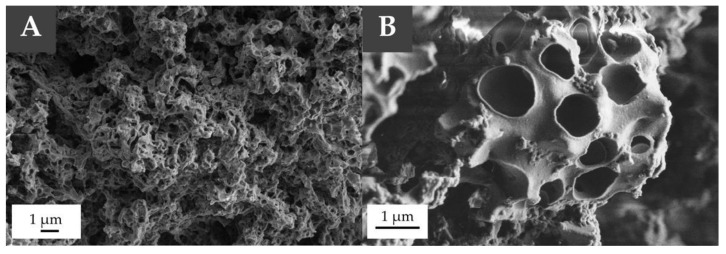
SEM pictures of a representative freeze-dried G1-polyHIPE crosslinked with 50 µl of GLU, (**A**) G1 (5K×); (**B**) G1 10K×.

**Figure 4:**
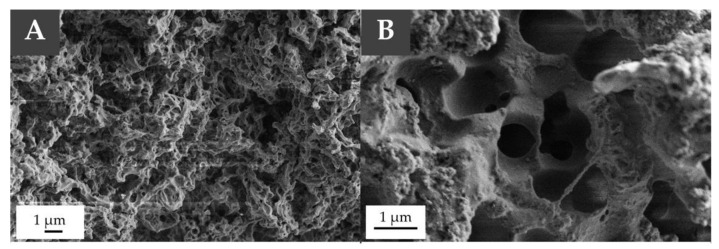
SEM pictures of a representative freeze-dried I-Series (I2) polyHIPE: (**A**) I2 (5K×); (**B**) I2 10K×.

In Scheme 1, the main activities resulting in the final solid polyHIPEs are summarized. From left to right, (1) HIPE formation (Figure 1 and Figure 2, A and B); (2) polyHIPE formation, i.e., crosslinking of the aqueous phase containing the polymers dispersion (Figure 1 and Figure 2, C); and (3) final polyHIPE after purification and lyophilization (Figure 3 and Figure 4). In particular, Figure 1 and Figure 2B show the qualitative demonstration of the o/w emulsion formation; in fact, the detection of the darker droplets of the oil phase, coloured by the introduction of Sudan Red G, could be noted.

Changing the amount of crosslinker in G-series HIPEs to 100 or 200 µL did not significantly change the main internal morphology of the final polyHIPEs.

The obtained polyHIPEs from GCS or INU-MA were characterized by morphological analysis to study their internal structures.

SEM of G1 polyHIPE, obtained by the crosslinking reaction with the minimum used amount of GLU (50 µl), showed a material with a alveolar-like structure and with an internal morphology characterized by homogeneously distributed voids, often interconnected by small throats. The main structure looks like a soft material due to the polysaccharide nature of the main component, see Figure 3. As shown in an exhaustive review by Silverstein, hydrogel-based polyHIPEs often exhibit an internal structure that is not as ordered as the W/O systems often are [27], as well as a reduced number of throats, which is probably due to the more flexible nature of polysaccharides with respect to other materials such as acrylates or styrene [12,28,29]. This is mostly linked to the fact that the throats are formed upon shrinkage of the polymer film surrounding the disperse phase during polymerization and this is particularly evident in polyacrylates or polystyrene [7,25]. On the other side, chitosan based polyHIPEs were previously prepared, showing a very similar internal structure [30]. Interestingly, a similar relatively low amount of throats in the internal structure was reported in another paper, which used chitosan for the formation of inverse polyHIPEs [31].

Figure 4 shows the internal structures of a representative polyHIPE produced from methacrylated INU. It can be noted from Figure 4A that the main internal structure shows numerous voids with small throats, as shown in Figure 4B, and also a partially collapsed structure, which is probably due, as mentioned before, to the softness of the polysaccharide. The pores (voids) and interconnections (throats) formed by the HIPE templating have the fundamental role of allowing gas and mass transfer between the material and the surface of contact. For example, permeability to oxygen would be fundamental for the wound healing process [32]. In a recent paper, it was demonstrated that konjac glucomannan films formulated as HIPE significantly increased their permeability to oxygen [33]. On the other side, the mass transfer would allow, for example, an optimal transfer of the liquids from the tissue to the hydrophilic material.

To evaluate the ability of these polyHIPEs to absorb and retain large amounts of water, i.e., body fluids, swelling studies were carried out in water at 37 °C. This is an important feature to be addressed since the polyHIPEs produced here could be proposed in a variety of different biomedical applications, especially in wound dressing, and for the controlled release of selected drugs.

**Figure 5:**
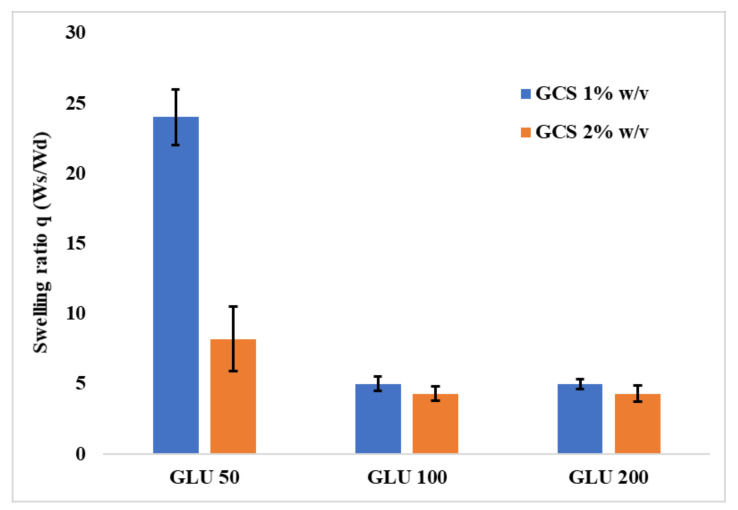
Swelling studies in water of G-series polyHIPEs (*n* = 3 for each sample, values are expressed ± SD).

Figure 5 reports the swelling ratio of the G-series polyHIPEs crosslinked with different amounts of GLU and at two different concentrations of GCS, namely 1% (G1) or 2% (G2) *w*/*v*.

As shown, the ability of these GCS-based materials to absorb water is both dependent on polymer concentration and the amount of crosslinker. This dual dependence is particularly evident when the smallest amount of GLU was used, namely 50 µl. In this case, G1 polyHIPEs had a q value of 24, while the q value was only 7 for G2. In the less concentrated dispersion, it could be assumed that the polymer chains are far each other and then, GLU will mostly determine an intra-chain crosslinking and a reduced inter-chain one, thus leading: (i) to the formation of the hydrogel (in fact, the product is insoluble in water, which is different to the starting GCS, which is dispersed in water), (ii) to a lower degree of crosslinking, which allows for pronounced swelling. By increasing the amount of GLU to 100 and up to 200 µl, a “plateau” in swelling is reached, thus indicating that a complete crosslinking was reached. This experimental evidence brings us to the conclusion that the swelling behaviour of such inverse polyHIPEs could be varied by varying the GCS concentration and/or GLU amount depending on the final needs. Interestingly, the variation of these parameters does not affect the HIPE (starting emulsion) stability.

**Figure 6:**
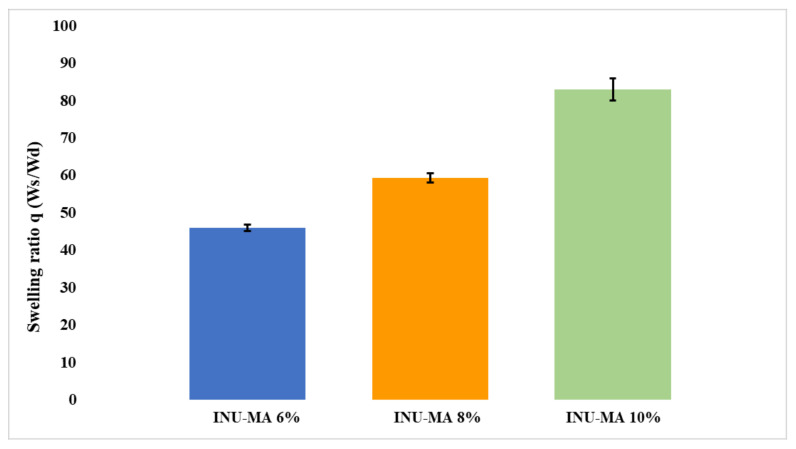
Swelling studies in water of I-series polyHIPEs (*n* = 3 for each sample, values are expressed ± SD).

Regarding I-series polyHIPE, as shown in Figure 6, the swelling is dependent on the polymer concentration. In particular, by increasing the polymer concentration from 6% to 10% *w/v*, the q values increased from 46 to 83 and was 59 for the concentration at 8% *w*/*v*. It should be noted that increasing the INUMA concentration did not result in macroscopic changes in the dispersion viscosity; at the same time, the stability of the produced HIPE was unaltered. Thus, I-series showed, differently from G-series, a direct concentration/swelling dependence. This is attributable to the different mechanism of crosslinking. In fact, in methacrylated INU, the netpoints starting from the methacrylic groups upon UV irradiation are homogeneously distributed within the polymer chain; thus, at the lower concentrations of polymer, these netpoints will be very close to each other, creating a more rigid system that will swell less [34,35]. This is due to a 3D development of the network that can bring different methacrylic groups to react closely, forming a stacked-like structure [36]. When the polymer concentration is higher, the radical crosslinking could even involve methacrylic groups from different polymer chains, therefore leading to a network with larger meshes and thus, explaining the higher q values by increasing the polymer concentration [37]. Overall, I-series showed q values much higher than G-series, thus further underlining the versatility in the production of inverse polyHIPEs by using these polysaccharides.

## 4. Conclusions

Here, we reported one of the few examples in the literature that shows the feasibility of inverse polyHIPEs materials by using polysaccharides in the aqueous phase in a *o*/*w* system. The materials were templated by the inverse HIPE technique comprising an oil internal phase at 80% *v*/*v*. The resulting materials showed a characteristic internal structure and a modulable swelling by changing the crosslinker amount and/or polymer concentrations. In this way, both GCS and INUMA were able to form the starting HIPE, which was stable, and even change the polymer concentrations of the emulsion. These materials were specifically designed for biomedical applications and in particular, for wound dressing application as medical devices and/or for drug release of entrapped active principles. Due to the hydrophilic nature of the external phase, the loading of peptides or proteins is predictable and easier to accomplish with respect to hydrophobic systems. This approach surely presents new perspectives regarding the production of inverse polyHIPEs.

## Figures and Tables

**Table 1:** Preliminary studies for high internal phase emulsion (HIPE) formation and stability of HIPEs from isopropyl myristate as the oil phase and *o*/*w v/v* ratio of 80%, using surfactants with different Hydrophilic Lipophilic Balance (HLB) and molecular weights (Mw).

Surfactant/HLB/Mw	Surfactant % *w*/*v*	Emulsion Formation	Emulsion Stability Time
**Tween 20/16.7/1228 Da**	10	no	-
	15	no	-
	20	no	-
	25	yes	5 min
	30	yes	15 min
**Lutrol F127/23/12600 Da**	10	no	-
	15	yes	>24 h
	20	yes	>24 h
	25	yes	>24 h
	30	yes	>24 h

**Table 2:** HIPE compositions with water as the aqueous phase, isopropyl myristate as the oil phase, and with an oil/water ratio of 80% *v*/*v*.

Sample	HIPE Code	Polymer % *w/v* in Aqueous Phase	Lutrol F127 % *w/v* in Aqueous Phase	Crosslinking Agent
G-series	G1	1	15	GLU *
	G2	2	15	GLU *
I-series	I1	6	15	UV light
	I2	8	15	UV light
	I3	10	15	UV light

* Glutaraldehyde (GLU) was added at three different concentration of 50, 100, or 200 µL for each G-series G1 and G2, therefore 6 polyHIPEs materials were obtained.
